# *Ex vivo* Evaluation of a New Drill System for Placement of Percutaneous Bone Conduction Devices

**DOI:** 10.3389/fsurg.2022.858117

**Published:** 2022-03-21

**Authors:** Ruben M. Strijbos, Louise V. Straatman, Robert J. Stokroos, Martin L. Johansson

**Affiliations:** ^1^Department of Otorhinolaryngology, Head and Neck Surgery, University Medical Centre Utrecht, Utrecht, Netherlands; ^2^University Medical Centre Utrecht Brain Centre, University of Utrecht, Utrecht, Netherlands; ^3^Department of Biomaterials, Institute of Clinical Sciences, Sahlgrenska Academy, University of Gothenburg, Gothenburg, Sweden; ^4^Research and Technology, Oticon Medical AB, Askim, Sweden

**Keywords:** dura, BAHS, bone drilling, osteotomy and drilling guides, bone anchored hearing, surgical procedures, bone conduction, minimally invasive

## Abstract

The procedure for installation of a percutaneous bone-conducting device has undergone significant improvements since its introduction 40 years ago. Today, the linear incision technique with tissue preservation (LITT-P) and the minimally invasive procedure (MIPS) are the most commonly used approaches. In both these techniques, a gradual increase of the osteotomy using a three-step drilling sequence is utilized, as this approach can allow a stepwise deepening and widening of the osteotomy in the mastoid and can prevent bone overheating. A new minimally invasive procedure (MONO) has been developed that allows an osteotomy to be performed and enables complete removal of the bone volume in one single drill step for a 4 mm implant using a novel parabolic twist drill. Here, the feasibility of the MONO procedure was qualitatively and quantitatively evaluated in terms of the dura response to drill trauma in comparison with the outcomes achieved with guide drills used for the LITT-P and MIPS techniques. Fresh frozen temporal bone from a human cadaver was subjected to penetration by three drills beyond the base of the mastoid bone to different depths. The sites were evaluated, and the damage to and possible penetration of the dura were determined. The results showed that for a drill depth exceeding mastoid bone thickness by not more than 1 mm, damage to the dura was limited or nonexistent, whereas for a drill depth exceeding bone thickness by 2 mm, damage increased, or the dura was penetrated. There was a trend toward more damage and penetration for both the round burr and MIPS guide drill compared with the MONO drill bit. From this experimental *ex vivo* study, it can be concluded that if the dura is encountered, the MONO system is not more inclined to penetrate the dura than the conventional LITT-P and MIPS systems.

## Introduction

In recent decades, the dynamic field of percutaneous bone conduction devices (BCDs) has inspired researchers from around the world to improve these implants, their abutment designs and related surgical techniques. These osseointegrated BCDs were introduced by Tjellström in 1977 and were based on the principle of bone conduction hearing (1). Currently, many people with hearing problems benefit from percutaneous BCDs, which is indicated for patients with uni- and bilateral conductive or mixed hearing loss (with an inability or intolerance to wear conventional hearing aids) (2, 3) and patients with single-sided deafness (4).

The related surgical technique has evolved from a free retroauricular full-thickness skin graft (5) to pedicled grafts, the dermatome technique and a linear incision technique with tissue reduction (LITT-R) (6–8). Although surgery was safe, adverse soft tissue reactions occurred (6–9). This led to the development of less invasive surgical approaches where LITT-R was modified to a linear incision technique with soft tissue preservation (LITT-P) (10), leading to reduced soft tissue-related problems (11–15). Moreover, more favorable results in terms of surgery time, cosmetic appearance and skin sensibility loss were registered (12, 15–19). Following the successful introduction of LITT-P, research has focused on further reducing the invasiveness of the surgical technique, e.g., by employing so-called punch-only techniques (20–23). A further refinement of the punch-only technique is the minimally invasive Ponto surgery (MIPS), introduced by Oticon Medical AB (Askim, Sweden) in 2015. Here, the implant is installed using a standardized surgical kit including a cannula that is used as a guide during the drilling sequence (24).

The results of the MIPS technique are encouraging, and several recent studies have compared the clinical outcome of MIPS with that of the commonly used LITT-P (25–29). While both techniques show favorable soft tissue outcomes compared with the outcomes of previous tissue reduction approaches, MIPS is also associated with improvements in terms of surgery time, cosmetics and preservation of skin sensibility (25–31). In addition to providing benefits to patients, improvements in clinical efficiency using MIPS have been reported in cost analysis studies (32). While several studies have reported comparable implant survival rates for MIPS compared to LITT-P (27–29, 31, 33), lower implant survival for MIPS has also been reported (25, 26, 34). Possible reasons can be the reduced visibility during the procedure, introducing a potential risk of angulated insertion or interposing soft tissue. Additionally, as a result of the smaller incision and guided drilling, there is a potential risk for excessive heat generation followed by negative effects on osseointegration at the implant site (35). Since the introduction of MIPS, the drill components in the surgical kit have been updated with the aim of reducing the heat generated during drilling (36). A recent clinical study reported a trend toward an improved implant survival rate using this updated MIPS system in comparison with the original drill system (37).

**Figure 1:**
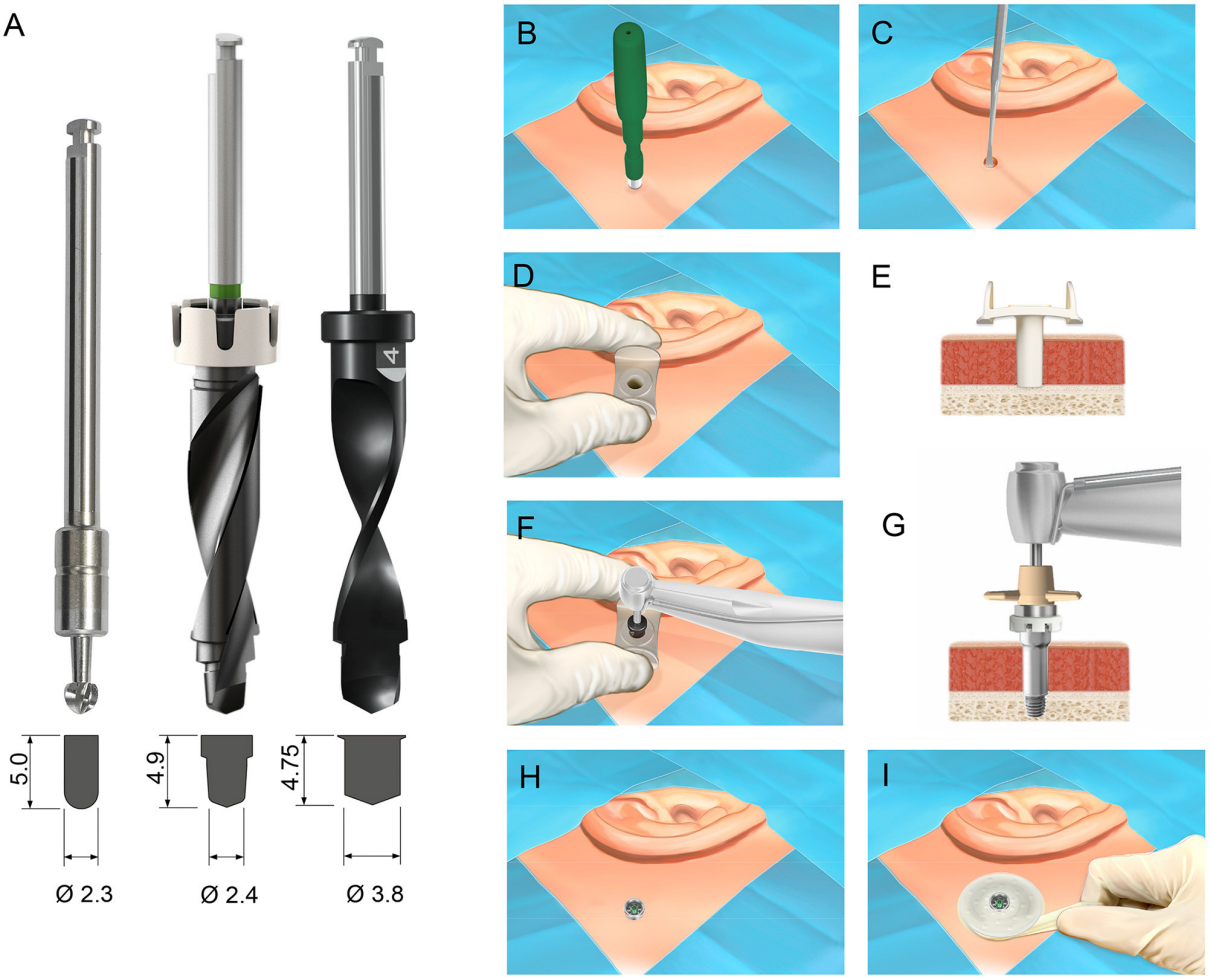
**(A)** The three drill bits evaluated in the study: the guide drill used the Ponto linear incision technique (left), MIPS guide drill (middle) and MONO drill (right). Below each drill, the shape of the osteotomy site following the drilling sequence using the respective drill is shown. Measurements in millimeters. **(B–I)** The surgical protocol for implantation of a percutaneous bone conduction device using the MONO procedure. **(B)** The skin is incised with a 5 mm biopsy skin punch. **(C)** The periosteum is removed from the bone surface at the site. **(D,E)** The cannula is inserted in the circular incision. **(F)** Osteotomy is created in one single drill step using the MONO drill. **(G,H)** The cannula is removed, and the implant is installed. **(I)** A healing cap and dressing are applied. Images courtesy of Oticon Medical AB © 2021.

Similar to the linear incision technique, MIPS employs a three-step drilling protocol with initial penetration using the cannula guide drill to a depth of 3.9 mm (38). If bone is present in the bottom of the osteotomy site, an additional 1 mm depth is created with the cannula guide drill, allowing the subsequent installation of a 4 mm long implant. To further optimize and simplify the drilling procedure, a novel drilling system, called the MONO procedure, has been developed in which the final osteotomy for a 4 mm implant is created in only one single drilling step, in contrast to the LITT-P and MIPS systems, where a three-step drilling sequence is employed (Figures 1A–I) (39, 40). In this single drilling step, the total drilling depth was 4.75 mm. The possible advantages of the MONO procedure are less drilling time and heat generation and fewer negative effects on peri-implant bone and osseointegration. In cases where the temporal bone thickness at the implantation site is less than the total drilling depth, the dura will be exposed and possibly traumatized or penetrated by the drill bit. Hence, a relevant and important prerequisite for the success of the MONO system is to evaluate the behavior of the drill bit when encountering the dura. The bone thickness in the area of the implantation site of BCDs has been evaluated in scientific studies (41, 42). Baker et al. reported average bone thicknesses of 6.78 ± 2.06 mm and 6.90 ± 2.27 mm (mean± SD) in adult patients with and without chronic ear disease, respectively (41). Kim et al. demonstrated that the average thickness was between 6.17 and 7.41 mm for patients aged 10 years or older. The study indicated that 95% of the adult population has a bone thickness of 5 mm or more in the area of the BCD position (42). Therefore, based on these evaluations, the MONO drill system, where the drill depth is 4.75 mm, can be considered a safe approach in an adult population with normal bone anatomy. In contrast, for the LITT-P and MIPS systems, the initial penetration of the mastoid bone is 4 mm and 3.9 mm, respectively, with corresponding final penetration depths of 5 and 4.9 mm when a 4 mm implant is installed. Therefore, using MONO, there is still a potentially higher risk for encountering the dura than with the LITT-P and MIPS systems.

The objective of the current study was to qualitatively and quantitatively evaluate the dura response to drill trauma using the MONO drill in comparison with the outcomes achieved with guide drills used for the LITT-P and MIPS techniques.

## Materials and Methods

### Study Design

**Figure 2:**
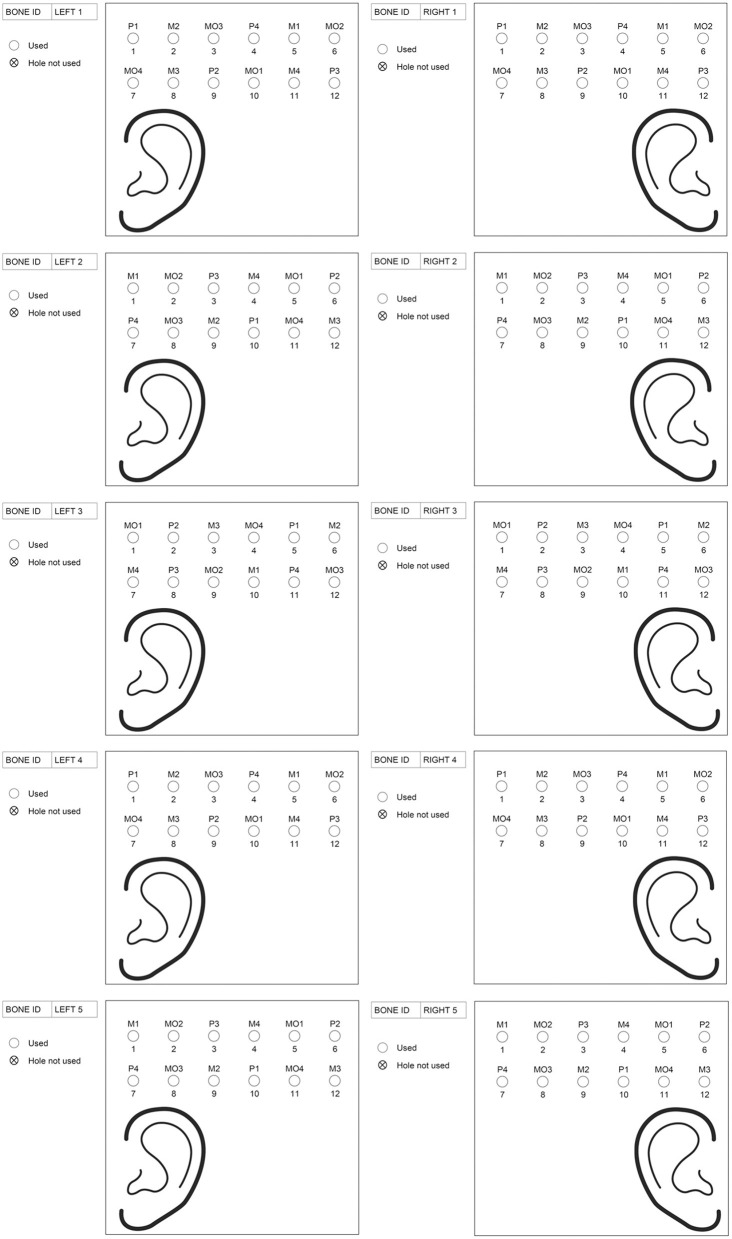
The predetermined randomized schedule with the drill sites for the different drills and penetration depths. The abbreviations used for the drilling systems are P = Ponto guide drill, M = MIPS guide drill and MO = MONO. The numbers behind the drilling systems indicate the penetration depth (e.g., P2 means Ponto guide drill with a penetration depth of 2.0 mm).

**Figure 3:**
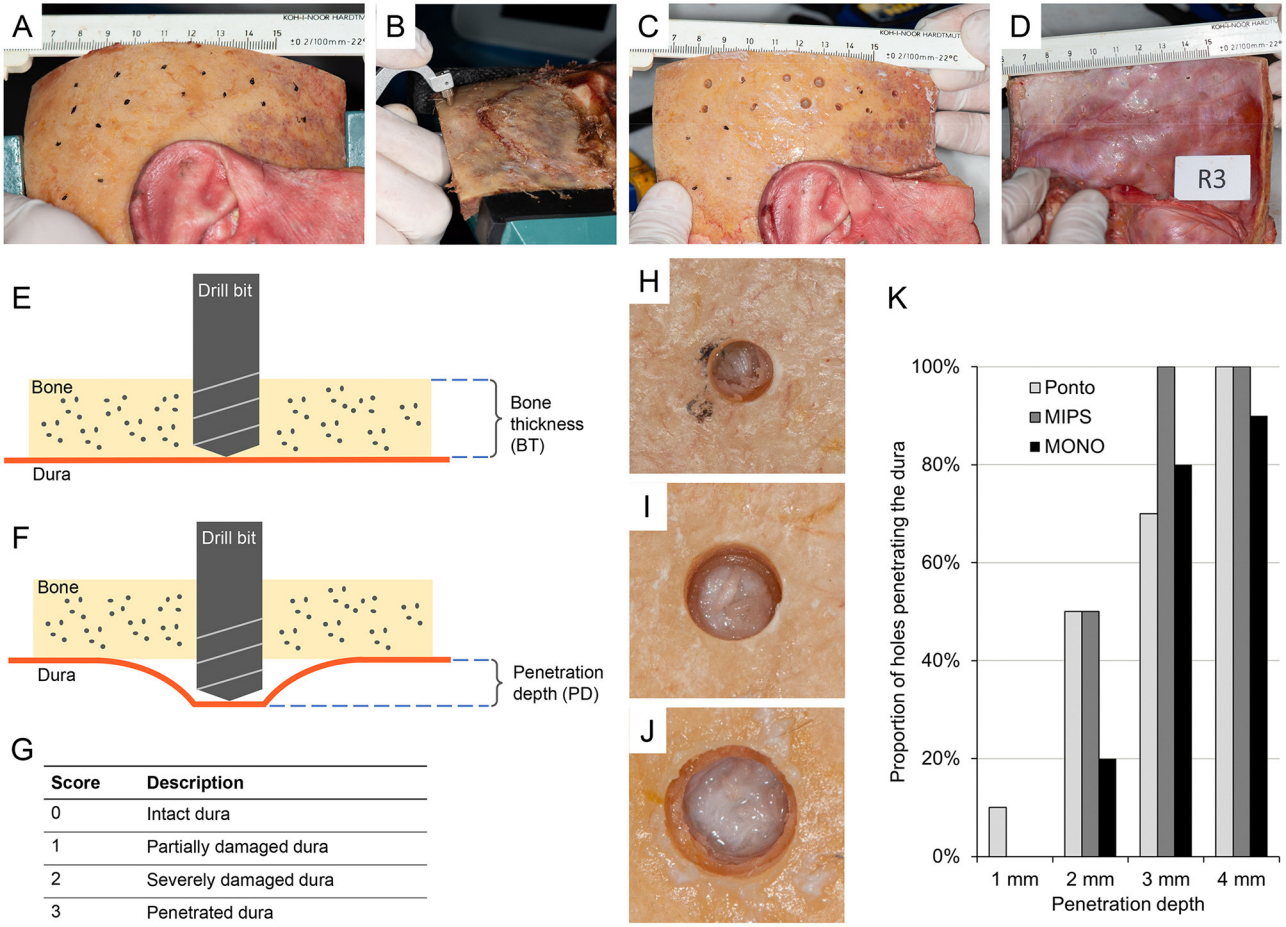
**(A)** Temporal bone sample (item R3) with removed soft tissue and sites marked according to the randomization scheme. **(B)** The bone thickness (BT) at the sites determined using a caliper. **(C)** Photograph of a temporal bone sample (item R3) after drilling sequence. **(D)** Photograph of the dura of the same temporal bone sample (item R3) after drilling was performed. **(E)** Illustration of the preparation of holes in the temporal bone. **(F)** Illustration of penetration depth. The drill bit penetrates beyond the base of the skull bone to different depths. **(G)** Scoring scale of the impact of the drill on the dural tissue. **(H)** Example of a hole using the round burr (Ponto) with a score of 2 indicating severely damaged dura. **(I)** Example of a hole using a guide drill (MIPS) with a score of 1 indicating partially damaged dura. **(J)** Example of a hole using a MONO drill with a score of 0 indicating intact dura. **(K)** Proportion of holes penetrating the dura for the different drill systems (Ponto, MIPS, MONO) and penetration depths (1, 2, 3 and 4 mm). If at least one of the inspector's scores signified penetration (a score of 3) for a specific hole, the dura was considered penetrated. *n* = 10 holes for each drill system, a and drill depth combination was prepared.

This was an *ex vivo* experimental study on cadaveric, fresh frozen, human temporal bone samples. Five temporal bone specimens were used with both right and left temporal bone samples (i.e., a total of ten temporal bone samples). The damage and possible penetration of the dura in these specimens were compared between drill bits and penetration depths. Three different drill bits were evaluated: the guide drill for the LITT-P technique (designated Ponto, P), the guide drill for the MIPS system (MIPS, M) and the MONO drill (MONO, MO) (Figure 1A). Penetration depth (PD) was defined as the depth of drilling beyond the base of the temporal bone (Figure 3B). To reduce the influence of variation in temporal bone sample and position on the results, the drill sites for the different drills and penetration depths were rotated using a predetermined randomized schedule (Figure 2). Two drill operators performed the experiment (MLJ and AH) with an equal distribution of temporal bone specimens between them.

### Test Procedure

The test procedure consisted of the following steps (Figures 3A–G):

- The temporal bone was identified, and soft tissue was removed.
- The drill sites were identified and marked according to the predetermined randomization scheme (Figure 2, Figure 3A), and the bone thickness (BT) at the sites was determined using a caliper (thickness gauge 2140–8105, domain 0–25 mm, accuracy 0.01 mm, Dasqua Tools, Cornegliano Laudense (LO), Italy) (Figures 3B,E). If a site could not be used for the stipulated test drill and test depth, the position was adjusted locally or moved to an alternative site on the bone.
- A drill site was selected, and a hole 0.5 mm shallower than the measured bone thickness was prepared using specially designed cannulas. The bottom of the hole was checked by visual inspection and palpation with a dissector. In the case of the presence of bone, a second cannula was used to drill deeper (with a maximum of 0.5 mm). When the dura was reached, this was defined as a “lower bone level” (Figure 3E).
- The drilling sequence against the dura was performed (Figures 3C,F). First, the drill was changed to a new one (i.e., a drill bit of the same type that is not used for hole preparation). Second, a new canula was used, which led to penetration depths of either 1.0, 2.0, 3.0 or 4.0 mm. As previously mentioned, the specific drill sites on the temporal bone were used to determine which penetration depth to use (according to the predetermined randomized schedule as outlined in Figure 2). Drilling was executed in a fast down-and-up motion while running the drill at 2 000 rpm using dental drill equipment (Drill unit SI-1023, Implantmed PLUS with hand piece WS-75 L, W&H Nordic AB, Täby, Sweden).
- The dura was inspected visually with a microscope (magnification x0.6 – x2.5, Zeiss OPMI Pico Surgical Microscope, Carl Zeiss AB, Stockholm, Sweden), and damage to the dura was scored according to a 4-point grading scale: 0 = intact dura, 1 = partially damaged dura, 2 = severely damaged dura and 3 = penetrated dura (Figures 3D,G). Four inspectors with expertise in the field of bone conduction devices (MJ, AH, MT and JL) independently scored each hole. If at least one of the inspector's scores indicated penetration (a score of 3) for a specific hole, the dura was considered to be penetrated. If all investigators scored 2 or below, the dura was considered not to be penetrated.

The test facility was PO Medica AB (Sparsör, Sweden), and the dates of the experiment were 30 September and 1 October 2019.

## Results

A total of ten fresh frozen temporal bones from five patients were used in the test. For each penetration depth and each drill system, ten sites were prepared. Hence, a total of 120 holes were made with 12 holes in each temporal bone specimen. Examples of dural impact after a drilling sequence with the three different drill bits can be seen in Figures 3H–J. Fourteen drill sites (12%), which were mostly located in the supra-auricular area and not in the region on the temporal bone of the (clinical) BCD implantation site, could not be used. In all these cases, the holes were moved to an alternative site on the same bone to conform with the study protocol. There was an equal distribution in alternative sites for holes between the different drill systems and penetration depths.

The complete results from the tests are presented in Supplementary Material 1, with the derived median and mean scores of each site shown in Table 1. At a penetration depth of 1.0 mm, none of the drill bits caused penetration of the dura, except in one case (10%) when the guide drill in the LITT-P system (designated Ponto guide drill) was used (i.e., one of the ten holes) (Figure 3K). At a 2.0 mm penetration depth, half of the cases penetrated the dura when using the Ponto or MIPS guide drills, whereas the dura was penetrated in only two cases (20%) using the MONO drill (Figure 3K). When drilling deeper to 3.0 and 4.0 mm beyond the inner bone level, 70% or more of the drill sequences caused penetration of the dura irrespective of the system (Figure 3K). The median and mean scores are in line with this trend. A sensitivity analysis with the cut-off threshold for dural penetration set to 2 points (i.e., the dura was considered penetrated if at least one inspector scored 2 or above) demonstrated a similar trend.

**Table 1:** The mean (± standard deviation) and median scores for damage to the dura with different drilling systems and penetration depths.

	**Mean score (±SD)**	**Median score**
P1	1.20 (0.69)	1
P2	2.25 (0.78)	2
P3	2.28 (1.01)	3
P4	2.70 (0.56)	3
M1	1.10 (0.67)	1
M2	2.25 (0.78)	2
M3	2.70 (0.61)	3
M4	2.68 (0.69)	3
MO1	1.08 (0.61)	1
MO2	1.63 (0.87)	2
MO3	2.70 (0.52)	3
MO4	2.73 (0.55)	3

*The dura scoring systems were graded as follows: 0, intact dura; 1, partially damaged dura; 2, severely damaged dura and 3, penetrated dura. The abbreviations used for the drilling systems are P, Ponto guide drill; M, MIPS guide drill and MO, MONO drill. The numbers behind the drilling systems indicate the penetration depth (e.g., P2 means Ponto guide drill with a penetration depth of 2.0 mm)*.

## Discussion

MIPS represents a promising minimally invasive, punch-only, surgical technique for BCD implantation. Comparable or improved soft tissue outcomes in combination with better results registered in surgical time, cosmetics, skin sensibility and in the field of cost analysis compared with the outcomes of traditional techniques can corroborate this statement (25–29, 32, 33, 37). These encouraging features make this technique relevant to improve and to further streamline the procedure to install a BCD. A new one-stage drilling procedure, called the MONO procedure (Oticon Medical), has been developed (39, 40). With this MONO drilling system, the final osteotomy site for a 4 mm implant is created in a single drill stage, in contrast to the available systems that employ a three-step drill sequence. It is possible that the reduced total drilling time and reduced heat generation may lead to less negative effects on the peri-implant bone and osseointegration (39, 40). Moreover, since some studies have reported lower implant survival with MIPS compared with LITT-P (25, 26, 34), developments leading to improved implant survival and stability outcomes using minimally invasive approaches are warranted.

In this *ex vivo* experimental study of human temporal bone samples, the novel MONO drill bit was compared with the guide drills of the LITT-P and MIPS systems in terms of damage and possible penetration of the dura. Interestingly, the MONO drill bit was less prone to inflict damage to the dura than the LITT-P and MIPS systems. Moreover, the MONO drill resulted in less penetration of the dura than the guide drills when the drill depth exceeded the mastoid bone thickness by 2 mm. A possible explanation for these findings could be related to the differences in the design of the drill bit tips. The tip diameters of the round burr in the LITT-P system and the MIPS guide drill are 2.3 and 2.4 mm, respectively. In contrast, the diameter of the MONO drill is 3.8 mm, resulting in a larger area of contact between the rotating drill tip and the dura tissue. In addition, the detailed design of the cutting edges differs between the three drill types. Another finding of the study was the fact that the dura is likely to be penetrated when the drill depth exceeds the mastoid bone thickness of more than 2 mm, irrespective of the drill system used. In a clinical situation, this would correspond to a mastoid bone thickness of <3.0 mm, 2.9 mm and 2.75 mm when using the LITT-P, MIPS and MONO systems, respectively.

Using the MONO procedure, the full depth (4.75 mm) of the osteotomy site was reached in a single drilling sequence, in contrast to the currently available systems where the drilling sequence is halted 1 mm before the full depth is reached to permit verification of bone tissue in the bottom of the osteotomy site before proceeding with the second drill step. Therefore, the impact of exposed dura during the drilling sequence may become more relevant. As stated in the introduction, previous research showed that 95% of adults have a bone thickness of 5 mm or more in the region of BCD implantation (42). This means that the MONO procedure should be considered a safe option for adult patients. However, the chance of encountering the dura is potentially more likely with this procedure than when using conventional systems. A recent systematic review showed that the mastoid bone is penetrated in ~6% of BCD surgeries (8). Obviously, a higher proportion could be expected in the pediatric population (43). There is, however, no indication that exposure of the dura would increase the complication rates in these patients. The penetration of the mastoid bone followed by penetration of the dura, with a resulting cerebrospinal fluid leak, is reported in the literature with a frequency of 0.3% of the cases, although without any serious adverse events reported in conjunction with this (8).

This experimental study has several strengths, some of which were present because the current test procedure was based on the fundaments and learning of two previous pilot studies. First, the localization on the temporal bone sample for the different test drills and test depths was randomly assigned. This led to a randomized predetermined scheme, which reduced possible effects on the results due to dissimilarity in temporal bone samples and position. Second, the temporal bones and dura of the samples were of good quality. This applied to all measurements because in case a site could not be used for the stipulated test drill and test depth according to scheme, e.g., because of insufficient quality of bone and/or dura, the position was adjusted locally or an alternative site on the bone was chosen. Third, an asset of the study was the ability to reach the level of the dura (i.e., lower bone level) precisely. This was accomplished by using a caliper to accurately identify the bone thickness before drilling. Additionally, this successful control of the drill depth was warranted by the implementation of step (iii) in the test procedure. This step consisted of preparing a hole using a cannula with a length of <0.5 mm compared to the measured bone thickness. This could be further deepened stepwise (in case the dura was not reached) using a second cannula with steps of <0.5 mm. A fourth strong facet of the experiment was the change to a new drill bit before drilling against the dura. This change prevented possible influences of instrumental wear on the drill bit. Another strength that should be considered was adequate documentation and photos of the temporal bone samples (Nikon D805 with AF-S Micro Nikkor 105 mm 2.8G ED). These are important factors for a clear and reproducible experimental study. Finally, grading was performed by professionals in the field of BCDs who scored the different drilled holes independently.

Nevertheless, some limitations of the study should be noted. First, the grading by observers was not fully blinded. Two of the four inspectors (MH and AH) performed both drilling and grading. Additionally, there was some considerable interobserver variability using our grading classification. Perhaps a more standardized approach/instruction for the inspectors could be useful. Another consideration may be the use of only one operator because subtle differences in drilling cannot be eliminated. Inevitable, interesting points were noted in the difference between “*in vivo*” tissue and cadaveric human temporal bone. However, these fresh frozen samples resemble “*in vivo*” outcomes better than artificial dura. Finally, it is important to recognize that in a clinical situation other side effects resulting from the drilling sequence may occur, e.g., bleeding from the bone and/or dura is commonly observed.

In conclusion, the novel MONO drilling procedure is not more inclined to penetrate the dura in cadaveric temporal human bone compared with drills used for the LITT-P and MIPS procedures. Based on the possible advantages of a one-step procedure for creating the osteotomy site, the MONO drilling procedure should be further developed, and its clinical useability should be evaluated.

## Data Availability Statement

The raw data supporting the conclusions of this article will be made available by the authors, without undue reservation.

## Author Contributions

MJ and RJS conceived, planned, and supervised the experiments. MJ, RMS, and LS processed the experimental data, performed the analysis, drafted the manuscript, and designed the figures. All authors provided critical feedback and helped shape the research, analysis and manuscript. All authors contributed to the article and approved the submitted version.

## Funding

This experimental study was supported by grants from Oticon Medical AB, Sweden, the Swedish Research Council (2018-02891, 2020-04715), the Swedish state under the agreement between the Swedish Government and the country councils (the ALF-agreement ALFGBG-725641), the Hjalmar Svensson Foundation, Adlerbertska Forskningsstiftelsen (GU 2019/86, 13/09/19), the IngaBritt and Arne Lundberg Foundation and the Area of Advance Materials of Chalmers and GU Biomaterials within the Strategic Research Area initiative launched by the Swedish Government.

## Conflict of Interest

MJ is a paid employee of Oticon Medical AB (Askim, Sweden). The remaining authors declare that the research was conducted in the absence of any commercial or financial relationships that could be construed as a potential conflict of interest.

## Publisher's Note

All claims expressed in this article are solely those of the authors and do not necessarily represent those of their affiliated organizations, or those of the publisher, the editors and the reviewers. Any product that may be evaluated in this article, or claim that may be made by its manufacturer, is not guaranteed or endorsed by the publisher.
