# Supplementary material for: Ex vivo Evaluation of a New Drill System for Placement of Percutaneous Bone Conduction Devices
Source: Front Surg. 2022 Mar 21;9:858117. doi: 10.3389/fsurg.2022.858117 (PMC8977416; doi:10.3389/fsurg.2022.858117)
Supplement: Supplementary file 1 [file Data_Sheet_1.DOCX]

Supplementary Material 1

Overview of the scoring of dura damage at the different drilling sites with different drilling systems and penetration depths. The bone sample (1 - 5 with left (L) and right (R)), different drill operators (MLJ and AH) and inspectors (MLJ, AH, MT and JL) are indicated. Used abbreviations for the drilling systems are P = Ponto guide drill (linear incision), M= MIPS guide drill and MO = MONO drill. The numbers behind the drilling systems indicate the penetration depth (e.g., P2 means Ponto guide drill with penetration depth 2.0 mm). Y/N indicates whether the dura was penetrated or not. If at least one investigator scored 3 (penetration) it was scored penetrated (=Y).

| Bone ID | L1 |  |  |  |  | R1 |  |  |  |  |
| --- | --- | --- | --- | --- | --- | --- | --- | --- | --- | --- |
| Drill operator | MLJ |  |  |  |  | AH |  |  |  |  |
| Investigator | MLJ | AH | MT | JL | **Penetrated?**  **Y/N** | MLJ | AH | MT | JL | **Penetrated?**  **Y/N** |
| P1 | 1 | 3 | 1 | 2 | **Y** | 2 | 1 | 1 | 1 | **N** |
| P2 | 3 | 3 | 3 | 2 | **Y** | 2 | 2 | 1 | 2 | **N** |
| P3 | 3 | 3 | 3 | 3 | **Y** | 2 | 1 | 1 | 2 | **N** |
| P4 | 3 | 3 | 3 | 3 | **Y** | 3 | 3 | 3 | 3 | **Y** |
| M1 | 2 | 2 | 2 | 0 | **N** | 2 | 1 | 1 | 0 | **N** |
| M2 | 2 | 2 | 1 | 1 | **N** | 3 | 3 | 3 | 3 | **Y** |
| M3 | 3 | 2 | 3 | 2 | **Y** | 3 | 3 | 3 | 3 | **Y** |
| M4 | 3 | 3 | 3 | 3 | **Y** | 1 | 3 | 0 | 2 | **Y** |
| MO1 | 2 | 1 | 1 | 0 | **N** | 2 | 1 | 1 | 0 | **N** |
| MO2 | 1 | 2 | 2 | 0 | **N** | 2 | 2 | 1 | 2 | **N** |
| MO3 | 3 | 3 | 3 | 3 | **Y** | 3 | 3 | 3 | 3 | **Y** |
| MO4 | 1 | 2 | 1 | 2 | **N** | 3 | 3 | 3 | 3 | **Y** |

| Bone ID | L2 |  |  |  |  | R2 |  |  |  |  |
| --- | --- | --- | --- | --- | --- | --- | --- | --- | --- | --- |
| Drill operator | AH |  |  |  |  | MLJ |  |  |  |  |
| Investigator | MLJ | AH | MT | JL | **Penetrated?**  **Y/N** | MLJ | AH | MT | JL | **Penetrated?**  **Y/N** |
| P1 | 1 | 1 | 0 | 0 | **N** | 2 | 2 | 2 | 2 | **N** |
| P2 | 3 | 2 | 3 | 3 | **Y** | 2 | 2 | 1 | 2 | **N** |
| P3 | 2 | 2 | 0 | 1 | **N** | 3 | 3 | 3 | 3 | **Y** |
| P4 | 3 | 2 | 3 | 2 | **Y** | 3 | 2 | 3 | 2 | **Y** |
| M1 | 1 | 1 | 0 | 1 | **N** | 1 | 2 | 1 | 1 | **N** |
| M2 | 3 | 3 | 3 | 3 | **Y** | 3 | 3 | 3 | 3 | **Y** |
| M3 | 3 | 3 | 3 | 3 | **Y** | 3 | 3 | 3 | 3 | **Y** |
| M4 | 3 | 2 | 3 | 3 | **Y** | 3 | 3 | 3 | 3 | **Y** |
| MO1 | 2 | 1 | 1 | 0 | **N** | 1 | 1 | 2 | 1 | **N** |
| MO2 | 2 | 2 | 0 | 1 | **N** | 3 | 2 | 3 | 3 | **Y** |
| MO3 | 3 | 3 | 3 | 3 | **Y** | 3 | 3 | 3 | 3 | **Y** |
| MO4 | 3 | 3 | 3 | 2 | **Y** | 3 | 3 | 3 | 3 | **Y** |

| Bone ID | L3 |  |  |  |  | R3 |  |  |  |  |
| --- | --- | --- | --- | --- | --- | --- | --- | --- | --- | --- |
| Drill operator | MLJ |  |  |  |  | AH |  |  |  |  |
| Investigator | MLJ | AH | MT | JL | **Penetrated?**  **Y/N** | MLJ | AH | MT | JL | **Penetrated?**  **Y/N** |
| P1 | 1 | 2 | 1 | 0 | **N** | 1 | 0 | 0 | 1 | **N** |
| P2 | 3 | 3 | 3 | 3 | **Y** | 3 | 3 | 3 | 3 | **Y** |
| P3 | 3 | 3 | 3 | 3 | **Y** | 1 | 2 | 3 | 2 | **Y** |
| P4 | 2 | 2 | 3 | 2 | **Y** | 3 | 3 | 1 | 3 | **Y** |
| M1 | 0 | 0 | 1 | 1 | **N** | 1 | 1 | 0 | 2 | **N** |
| M2 | 2 | 2 | 1 | 1 | **N** | 1 | 2 | 1 | 2 | **N** |
| M3 | 2 | 1 | 3 | 1 | **Y** | 1 | 2 | 3 | 3 | **Y** |
| M4 | 3 | 2 | 3 | 2 | **Y** | 3 | 3 | 3 | 3 | **Y** |
| MO1 | 1 | 0 | 1 | 1 | **N** | 1 | 1 | 1 | 0 | **N** |
| MO2 | 1 | 1 | 1 | 1 | **N** | 1 | 2 | 2 | 1 | **N** |
| MO3 | 3 | 3 | 3 | 3 | **Y** | 2 | 2 | 3 | 2 | **Y** |
| MO4 | 2 | 2 | 3 | 3 | **Y** | 2 | 2 | 3 | 3 | **Y** |

| Bone ID | L4 |  |  |  |  | R4 |  |  |  |  |
| --- | --- | --- | --- | --- | --- | --- | --- | --- | --- | --- |
| Drill operator | AH |  |  |  |  | MLJ |  |  |  |  |
| Investigator | MLJ | AH | MT | JL | **Penetrated?**  **Y/N** | MLJ | AH | MT | JL | **Penetrated?**  **Y/N** |
| P1 | 1 | 1 | 2 | 1 | **N** | 1 | 1 | 2 | 1 | **N** |
| P2 | 3 | 3 | 3 | 3 | **Y** | 1 | 2 | 2 | 2 | **N** |
| P3 | 3 | 3 | 3 | 3 | **Y** | 3 | 3 | 3 | 3 | **Y** |
| P4 | 1 | 3 | 3 | 3 | **Y** | 3 | 3 | 3 | 3 | **Y** |
| M1 | 1 | 0 | 1 | 1 | **N** | 2 | 2 | 2 | 1 | **N** |
| M2 | 2 | 2 | 2 | 2 | **N** | 2 | 3 | 3 | 3 | **Y** |
| M3 | 3 | 3 | 3 | 3 | **Y** | 3 | 3 | 3 | 3 | **Y** |
| M4 | 3 | 3 | 3 | 3 | **Y** | 3 | 3 | 3 | 3 | **Y** |
| MO1 | 2 | 1 | 1 | 1 | **N** | 2 | 2 | 2 | 1 | **N** |
| MO2 | 1 | 2 | 2 | 2 | **N** | 3 | 3 | 3 | 3 | **Y** |
| MO3 | 1 | 2 | 2 | 2 | **N** | 3 | 3 | 3 | 3 | **Y** |
| MO4 | 3 | 3 | 3 | 3 | **Y** | 3 | 3 | 3 | 3 | **Y** |

| Bone ID | L5 |  |  |  |  | R5 |  |  |  |  |
| --- | --- | --- | --- | --- | --- | --- | --- | --- | --- | --- |
| Drill operator | MLJ |  |  |  |  | AH |  |  |  |  |
| Investigator | MLJ | AH | MT | JL | **Penetrated?**  **Y/N** | MLJ | AH | MT | JL | **Penetrated?**  **Y/N** |
| P1 | 1 | 1 | 2 | 1 | **N** | 1 | 2 | 1 | 1 | **N** |
| P2 | 1 | 1 | 2 | 1 | **N** | 1 | 1 | 2 | 2 | **N** |
| P3 | 1 | 3 | 3 | 3 | **Y** | 1 | 0 | 1 | 0 | **N** |
| P4 | 3 | 3 | 3 | 3 | **Y** | 3 | 3 | 3 | 2 | **Y** |
| M1 | 2 | 1 | 1 | 1 | **N** | 1 | 2 | 1 | 1 | **N** |
| M2 | 3 | 3 | 3 | 2 | **Y** | 2 | 2 | 1 | 1 | **N** |
| M3 | 3 | 3 | 3 | 3 | **Y** | 2 | 3 | 3 | 2 | **Y** |
| M4 | 1 | 2 | 3 | 2 | **Y** | 3 | 3 | 3 | 3 | **Y** |
| MO1 | 2 | 1 | 1 | 0 | **N** | 1 | 1 | 1 | 1 | **N** |
| MO2 | 1 | 1 | 2 | 1 | **N** | 1 | 1 | 1 | 0 | **N** |
| MO3 | 3 | 3 | 3 | 3 | **Y** | 2 | 2 | 2 | 2 | **N** |
| MO4 | 3 | 3 | 3 | 3 | **Y** | 3 | 3 | 3 | 3 | **Y** |
